# Supplementary material for: ZFR coordinates crosstalk between RNA decay and transcription in innate immunity
Source: Nat Commun. 2018 Mar 20;9:1145. doi: 10.1038/s41467-018-03326-5 (PMC5861047; doi:10.1038/s41467-018-03326-5)
Supplement: Supplementary file 1 — Supplementary Information(PDF 16572 kb) [file 41467_2018_3326_MOESM1_ESM.pdf]

ZFR coordinates crosstalk between RNA decay and transcription in innate immunity

Haque et al.

Supplementary Figure 1

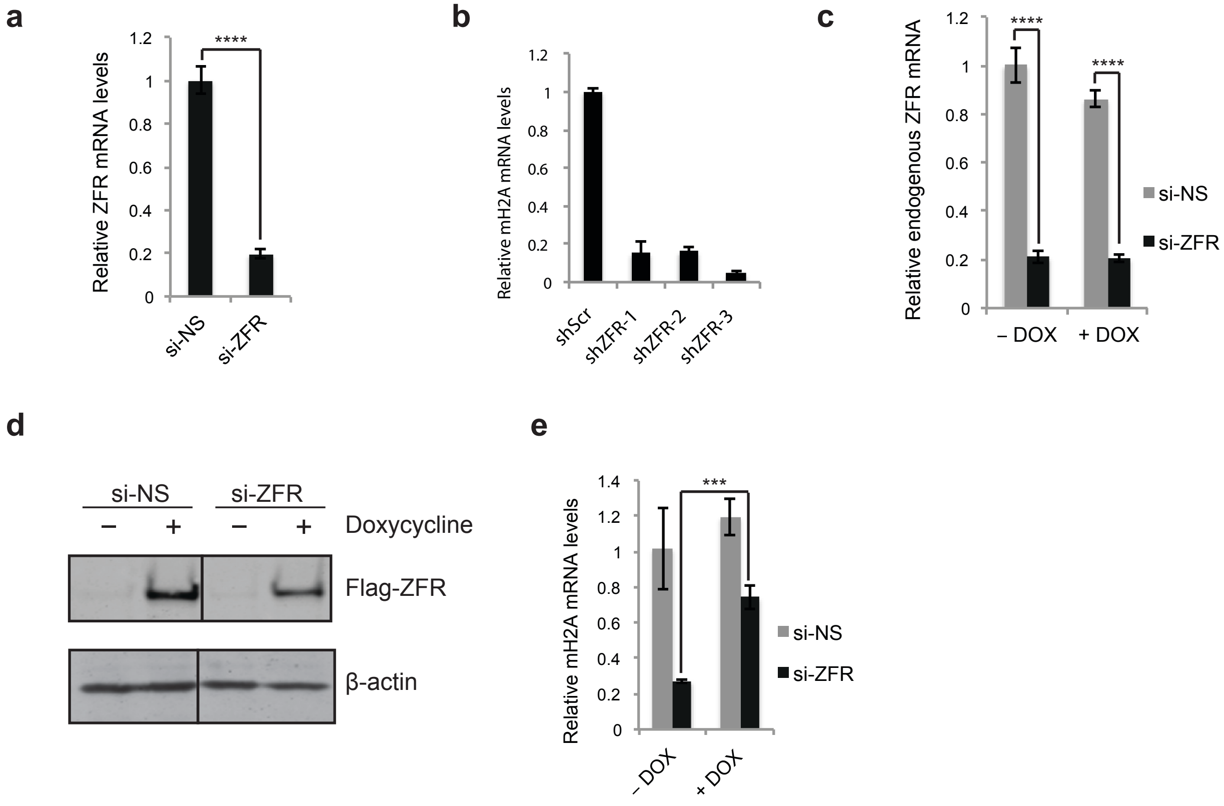

Supplementary Figure 1. ZFR controls mH2A1 expression.
(**a**) qRT-PCR analysis of ZFR in HEK-293TO cells transfected with siRNA against ZFR (si-ZFR) or non-silencing control siRNA (si-NS; n=3). (**b**) qRT-PCR analysis of mH2A1 in HEK-293TO cells stably transduced with three distinct shRNAs against ZFR (shZFR-1, shZFR-2, shZFR-3) or a non-targeting shRNA (shScr) (n=2). (**c**) qRT-PCR analysis using primers against the ZFR 3'UTR in HEK-293TO cells expressing doxycycline (DOX)-inducible siRNA-resistant 3xFLAG-tagged ZFR, following transfection with si-ZFR or si-NS (n=3). (**d**) Immunoblot analysis of 3xFLAG-tagged ZFR; β-actin was used as a loading control. Approximate MW based on size markers is shown. (**e**) qRT-PCR of mH2A1 in cells expressing a siRNA-resistant and doxycycline (DOX)-inducible ZFR cDNA, following transfection with si-ZFR or si-NS. Graphs indicate mean ± SD; *** $P<0.001$; **** $P<0.0001$ (two-tailed Student's t test).

Supplementary Figure 2

a

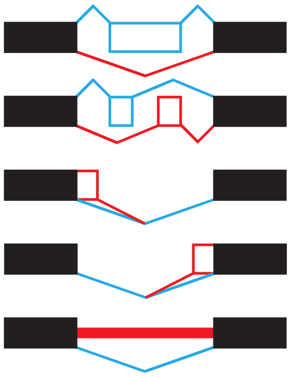

	Total AS events observed	Events affected by siZFR	% of observed events affected	Distribution vs. expected
Exon skipping/inclusion	11561	158	1.4	Enriched ($P = 0.0006$)
Mutually exclusive	2343	32	1.4	n.s. ($P = 0.22$)
Alternative 5' splice site	3154	26	0.8	n.s ($P = 0.10$)
Alternative 3' splice site	4537	23	0.5	Depleted ($P < 0.0001$)
Intron retention	3112	37	1.2	n.s. ($P = 0.65$)

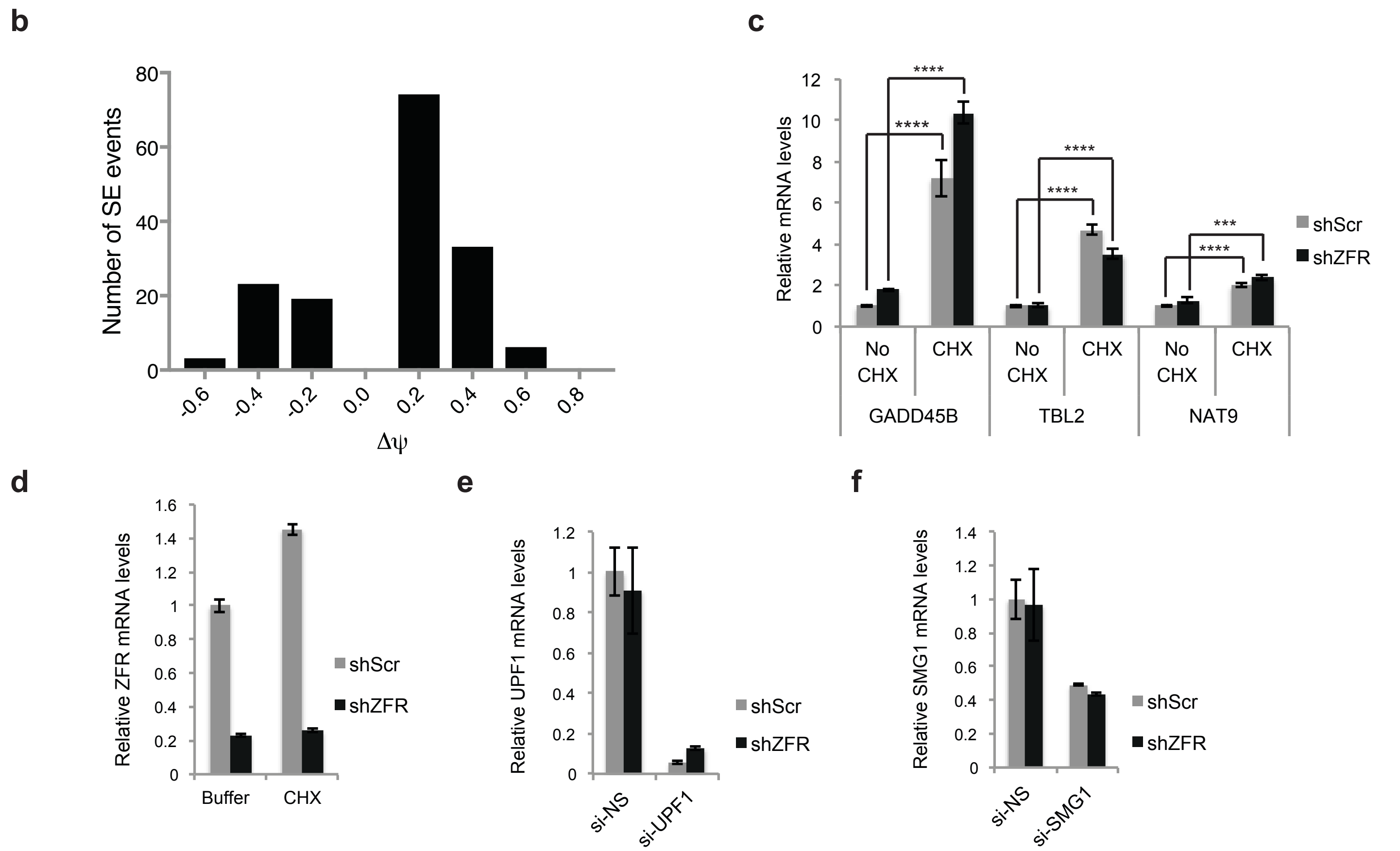

Supplementary Figure 2. ZFR regulates alternative splicing in HEK-293TO cells.
(a) Table of alternative splicing events affected by ZFR knockdown in HEK-293TO cells. Numbers of total alternative splicing events meeting read coverage cutoffs and events significantly changed by ZFR knockdown in MISO analysis (bayes factor > 20 and $|\Delta\Psi| > 0.12$) are indicated (see Materials and methods for details). (b) Histogram of distribution of exon inclusion (positive ΔΨ) vs exclusion (negative ΔΨ) events in HEK-293TO cells upon ZFR depletion. (c) qRT-PCR of mRNAs previously characterized as NMD substrates (GADD45B, TBL2, NAT9) with or without cycloheximide (CHX) treatment in shZFR or shScr cells, as in Fig. 2E. (d) qRT-PCR of ZFR mRNA in cells stably depleted of ZFR (shZFR) and control cells (shScr) with or without treatment with cycloheximide (CHX; n=3). (e) qRT-PCR of UPF1 in cells stably depleted of ZFR (shZFR) or control cells (shScr) transfected with siRNAs specific to UPF1 (si-UPF1) or a non-specific control (si-NS; n=2). (f) qRT-PCR of SMG1 in cells transfected with siRNAs against SMG1 (si-SMG1) or si-NS (n=2).

Supplementary Figure 3

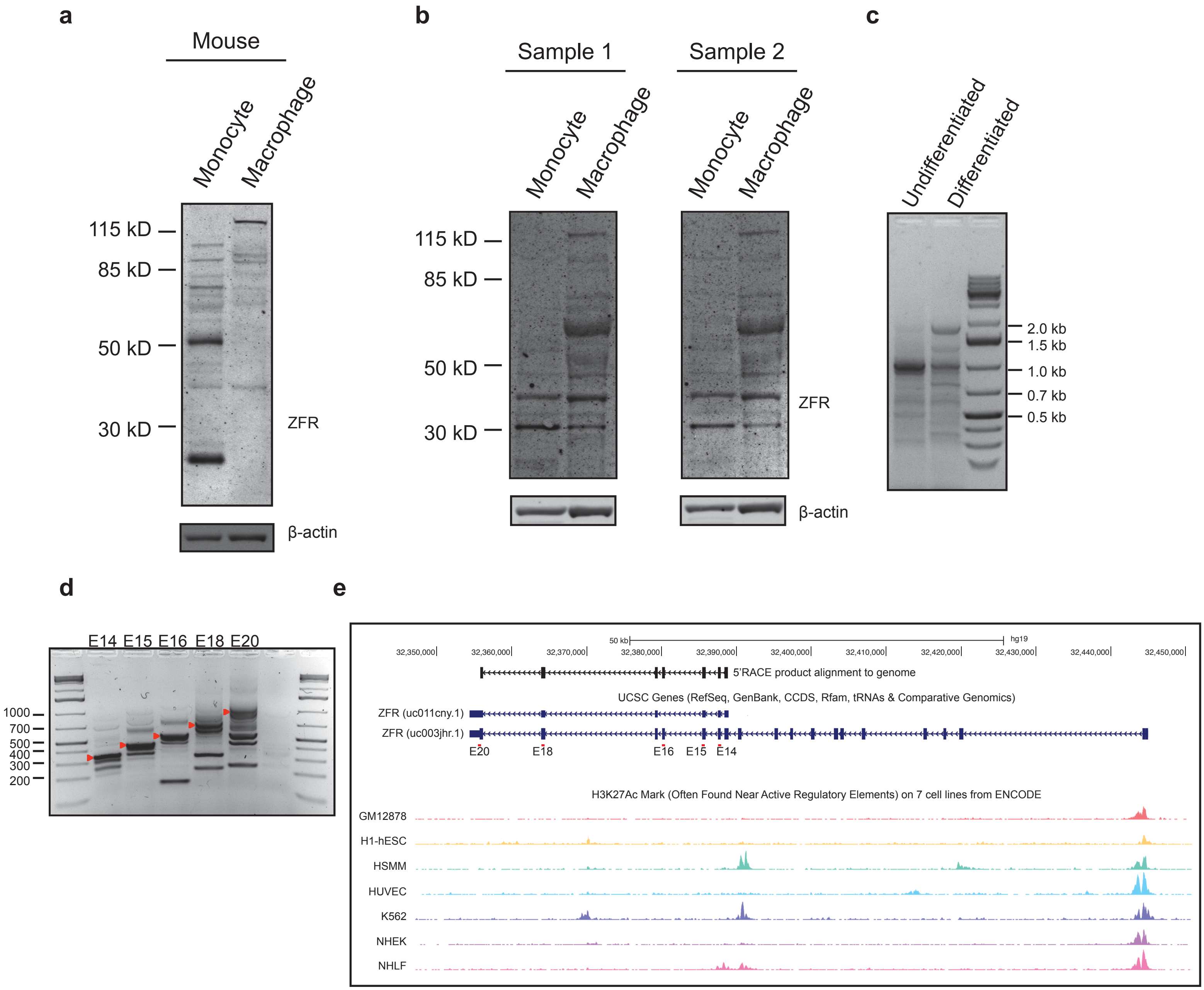

Supplementary Figure 3. Characterization of truncated ZFR isoform expressed in monocytic cells. (**a**) Immunoblot analysis of ZFR in murine BMDMs before and after differentiation. (**b**) Immunoblot analysis of human peripheral blood monocytes and monocyte-derived macrophages. β-Actin was used as a loading control. Approximate MW based on size markers is shown. (**c**) RT-PCR products from 5'RACE using total RNA from undifferentiated and differentiated THP-1 cells. (**d**) 5'RACE of ZFR mRNAs expressed in undifferentiated THP-1 cells. Primers in the indicated exons downstream of the previously annotated alternative 5' end (UCSC mRNA uc011cny.1) were used to amplify 5'RACE products. Arrows denote products corresponding to those subjected to Sanger sequencing.(**e**) Schematic of the ZFR locus depicting transcripts arising from alternative promoters. Top, positions of BLAT alignment of the truncated ZFR isoform sequence derived from 5'RACE products. Middle, UCSC gene models showing full-length ZFR (uc003jhr.1) and a transcript starting in exon 13 (uc011cny.1), as observed in 5'RACE. Note that uc011cny.1 lacks exon 16, which was retained in the 5'RACE products sequenced from THP-1 cells. Usage of an in-frame methionine in exon 15 is predicted to give rise to a polypeptide terminating at the canonical ZFR termination codon. Bottom, ENCODE ChIP-Seq data indicating cell type-specific enrichment of H3K27 acetylation near the standard and alternative ZFR promoters (ENCODE Project Consortium, 2012).

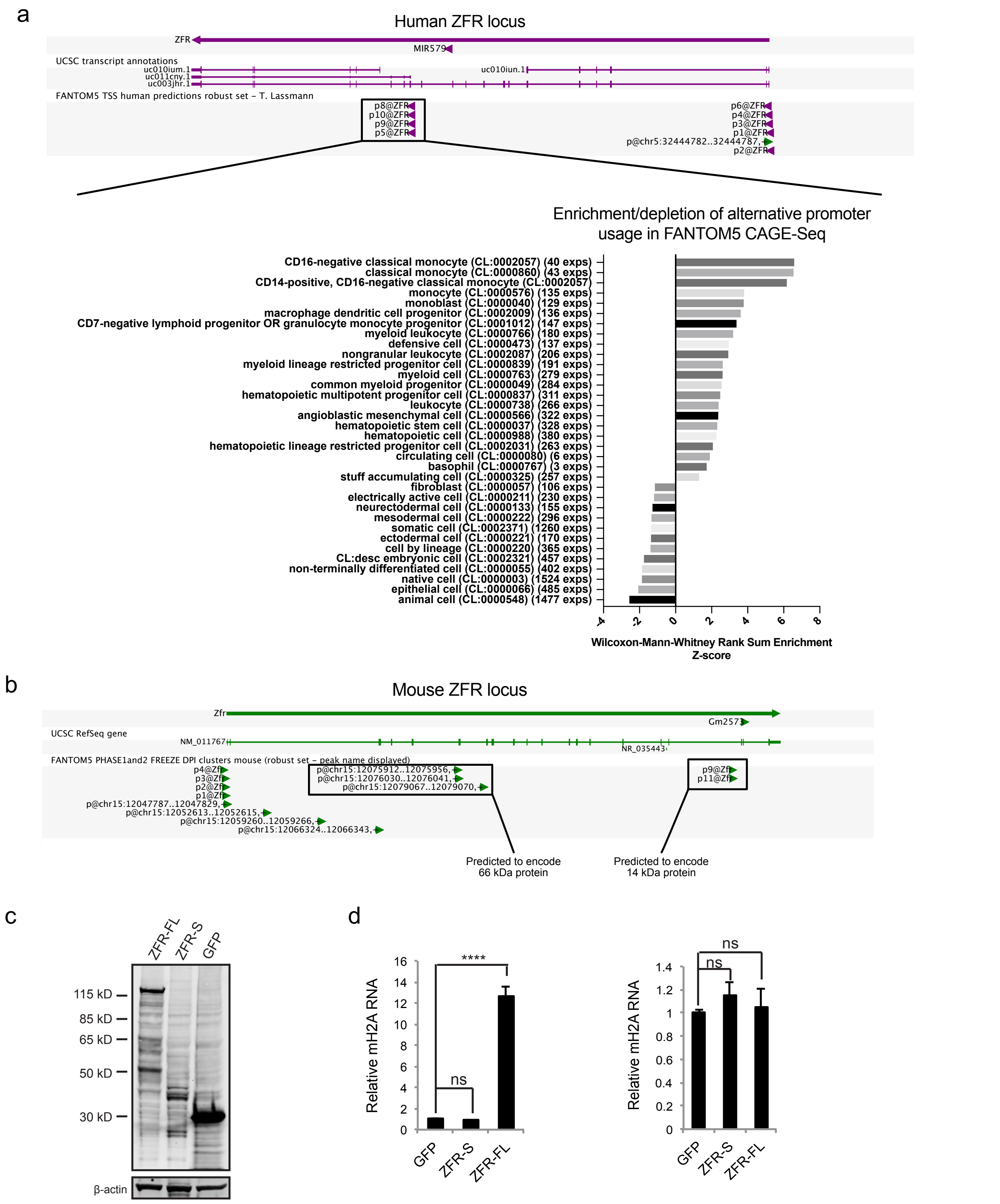

Supplementary Fig. 4 Differential ZFR promoter usage in human and mouse cells generates an inactive ZFR isoform
(a) Top, positions of ZFR transcriptional start sites, as determined by CAGE-Seq and visualized using the ZENBU genome browser (FANTOM consortium). Bottom, TSSs generating truncated ZFR were identified as preferentially used in primary monocytes and monocytic cell lines. (**b**) Positions of TSSs identified by FANTOM CAGE-Seq in mouse cells are indicated. (**c**) cDNAs from full-length ZFR and 5'RACE experiments conducted in monocytic THP-1 cells were transfected into HEK-293 cells. Approximate MW based on size markers is shown. (**d**) qRT-PCR of mH2A1 in ZFR-depleted cells (shZFR, left) and control cells (shScr, right) transiently transfected with either full-length (FL) or short (S) isoform of ZFR cDNA, and GFP cDNA as control. Graphs indicate mean ± SD; **** P<0.0001; ns, P>0.05 (two-tailed Student's t test).

Supplementary Figure 5

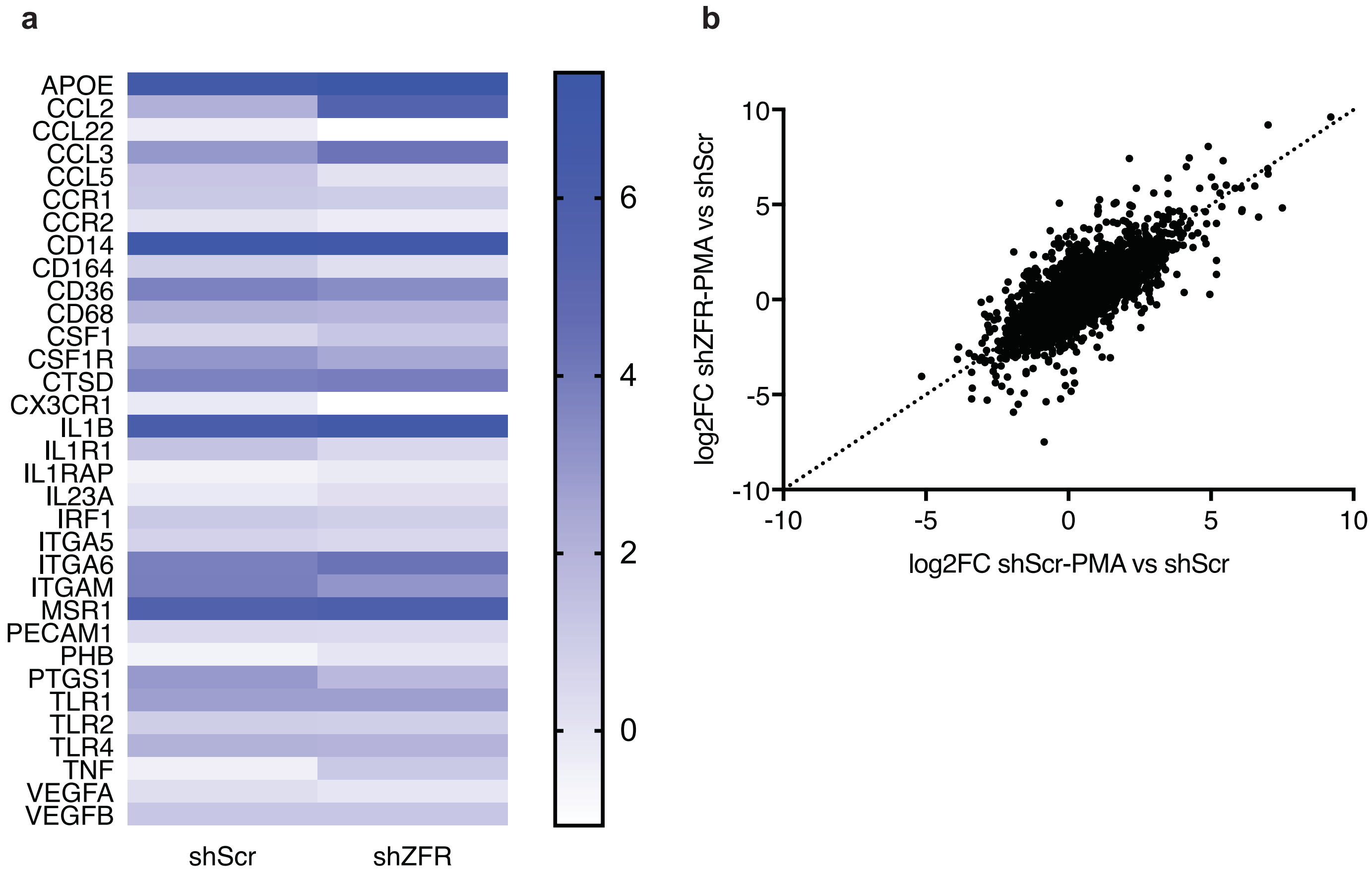

Supplementary Figure 5. THP-1 cells depleted of ZFR undergo differentiation into macrophage-like cells. (**a**) Heatmap of changes in expression of genes previously characterized as affected by differentiation of THP-1 cells with PMA [23]. Levels of the indicated genes in shZFR- and shScr-treated differentiated THP-1 cells were compared to levels in undifferentiated shScr THP-1 cells. (**b**) Scatterplot of gene expression changes upon differentiation of ZFR knockdown and control THP-1 cells. mRNA expression levels in differentiated shZFR- and shScr-treated THP-1 cells were compared to expression levels in undifferentiated shScr-treated cells. Dotted line indicates equal values in shZFR and shScr PMA-treated samples.

Supplementary Figure 6

a

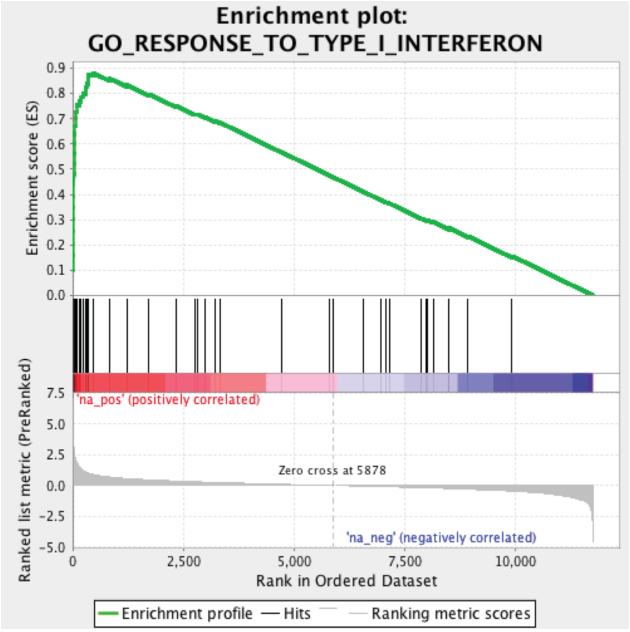

b

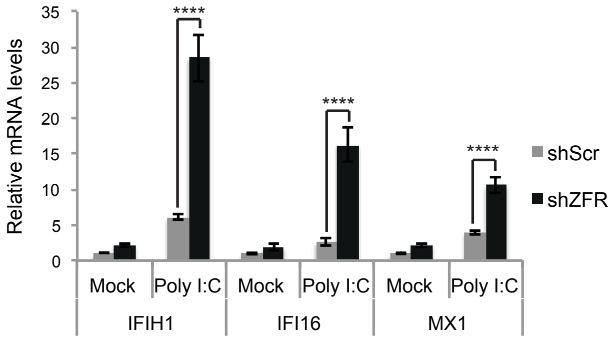

Supplementary Figure 6. ISGs are induced in THP-1 cells depleted of ZFR.
(**a**) Enrichment plot generated by Gene Set Enrichment Analysis (GSEA) software, using RNAseq data from PMA-treated ZFR knockdown and control THP-1 cells. GSEA indicates significant enrichment of ISGs among genes induced by ZFR depletion (P<0.001). (**b**) qRT-PCR analysis of IFIH1, IFI16, and MX1 expression in HEK-293TO cells treated with the indicated shRNAs as in Fig. 5F (n=4). Graph indicates mean ± SD; **** P<0.0001 (two-tailed Student's t test).

Supplementary Figure 7

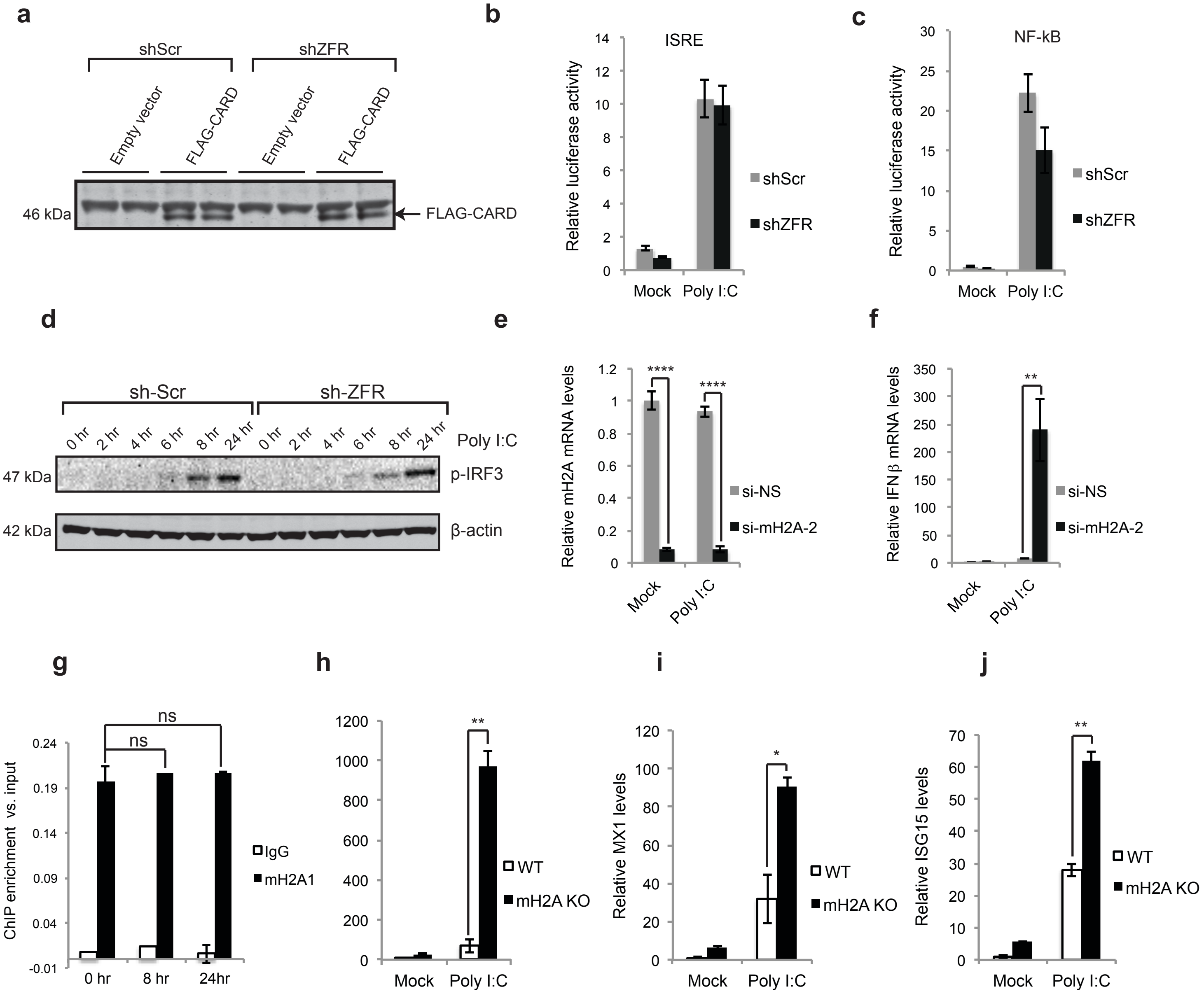

Supplementary Figure 7. ZFR inhibits IFNβ transcription downstream of MAVS and IRF3.
(**a**) Immunoblot analysis indicating equivalent expression of 3XFLAG-RIG-I-CARD in cells used for Fig 6A. (**b**) Dual luciferase assay using constructs harboring firefly luciferase driven by either five copies of interferon-stimulated response elements (ISRE; n=3) or (**c**) five copies of NF-kB binding sites (n=3). Graphs indicate mean ± SD; **P<0.01, ***P<0.001, ****P<0.0001 (two-tailed Student's t test). (**d**) Immunoblot analysis of phospho-IRF3 after stimulation of HEK-293TO cells with poly I:C. β-actin is used as a loading control. Approximate MW based on size markers is shown. (**e**) qRT-PCR analysis of mH2A1 in HEK-293TO cells transfected with a second siRNA specific to mH2A (si-mH2A-2) or si-NS and stimulated with poly I:C or buffer only (Mock; n=3). (**f**) qRT-PCR analysis of IFNβ in HEK-293TO (n=3). (**g**) Chromatin immunoprecipitation-qPCR (ChIP-qPCR) analysis of mH2A1 binding to the IFNβ promoter at different time points after stimulation with poly I:C. (**h**) qRT-PCR analysis of IFNα, (**i**) MX1, and (**j**) ISG15 in BMDMs isolated from either WT or mH2A double knockout mice (mH2A KO) and stimulated with poly I:C. Graphs indicate mean ± SD; ns P>0.05, *P<0.05, ** P<0.01, *** P<0.001, **** P<0.0001 (two-tailed Student's t test).

Supplementary Figure 8

Fig. 1D

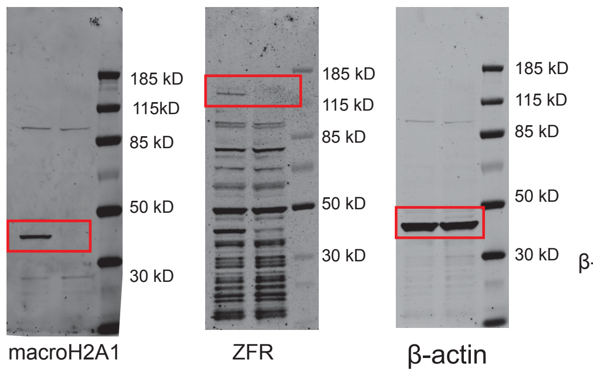

Supplementary Fig. 1D

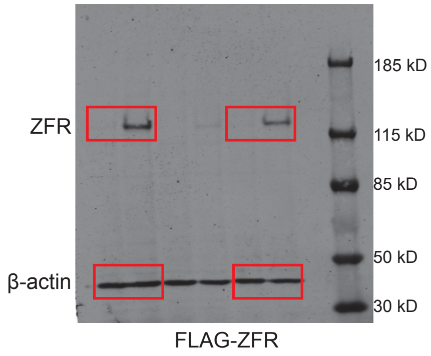

Fig. 3B

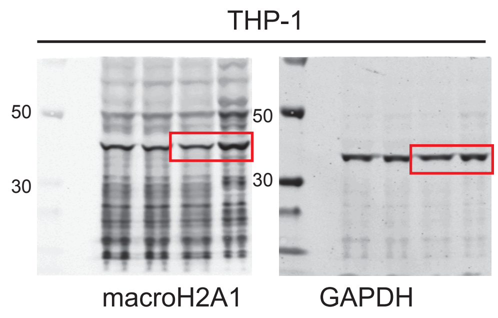

Fig. 3B

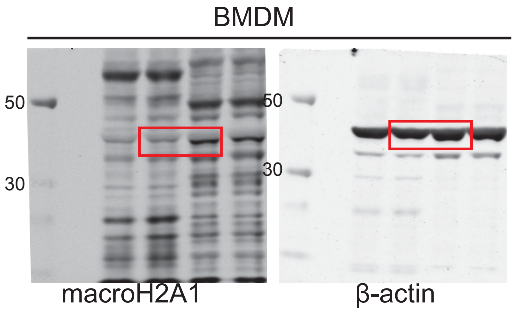

Fig. 3C

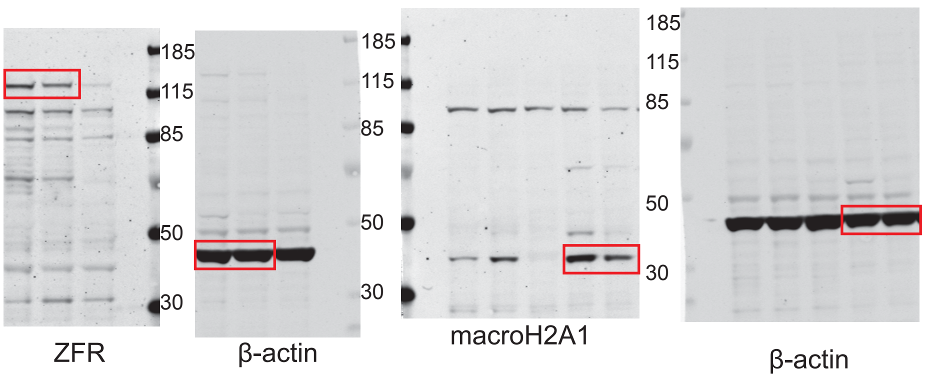

Fig. 6J

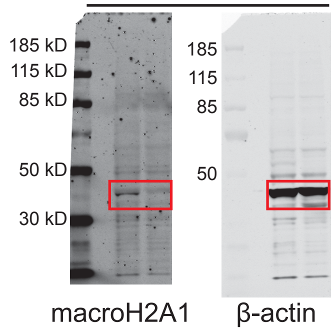

Supplementary Fig 7A

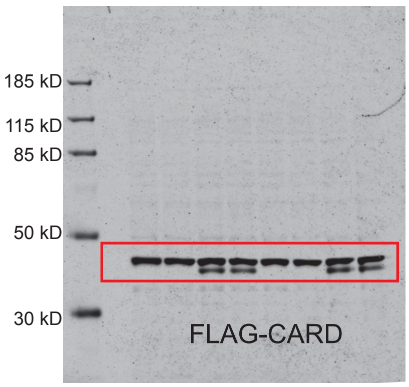

Supplementary Fig 7D

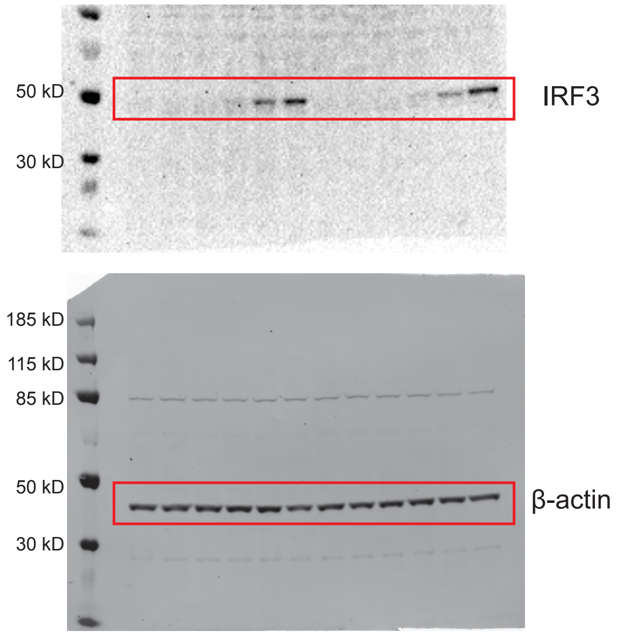

Supplementary Figure 8. Immunoblots

Supplementary Figure 9

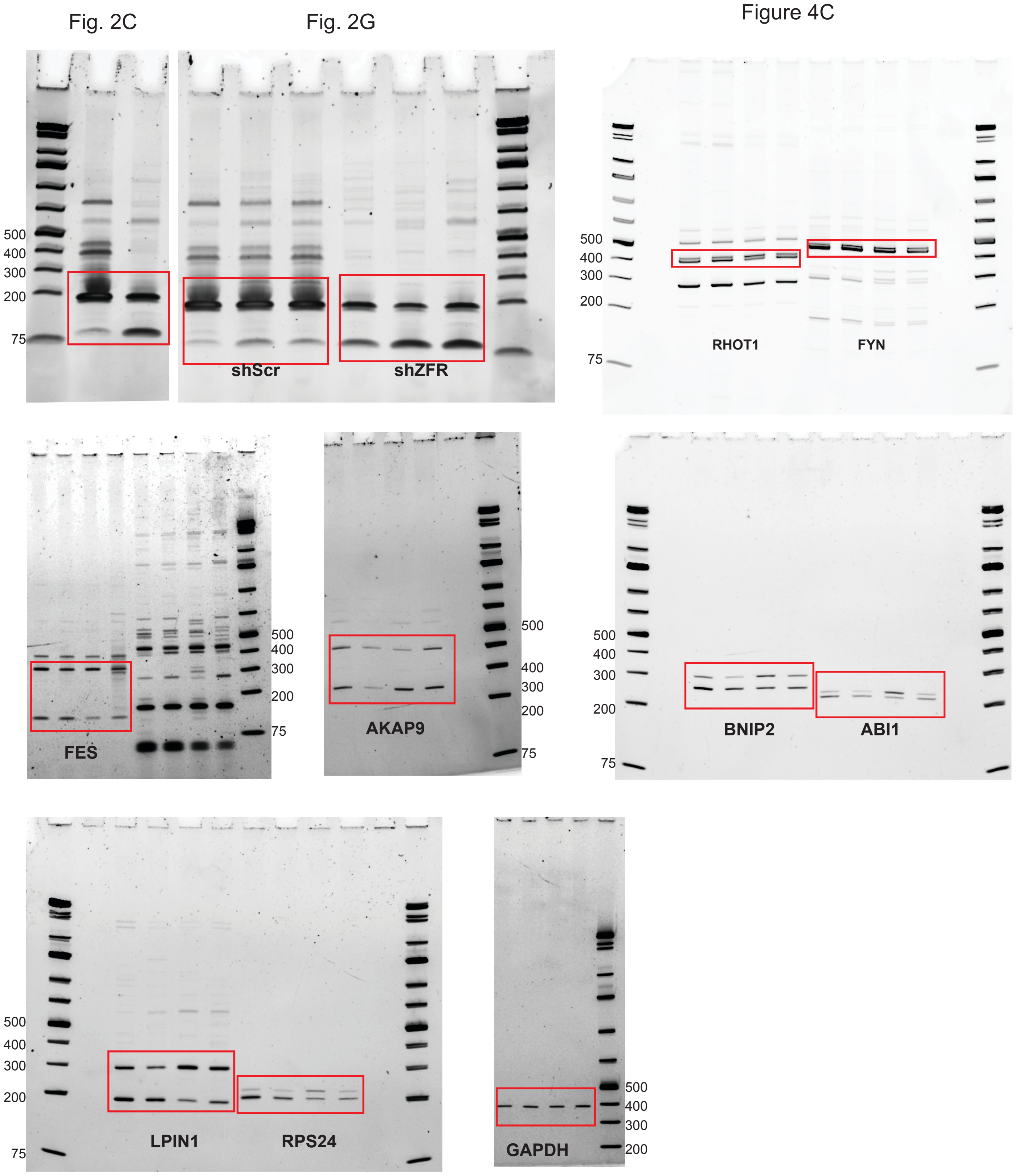

Supplementary Figure 9. RT-PCR gels
