# Supplementary material for: ZFR coordinates crosstalk between RNA decay and transcription in innate immunity
Source: Nat Commun. 2018 Mar 20;9:1145. doi: 10.1038/s41467-018-03326-5 (PMC5861047; doi:10.1038/s41467-018-03326-5)
Supplement: Supplementary file 3 — Description of Additional Supplementary Files(PDF 86 kb) [file 41467_2018_3326_MOESM3_ESM.pdf]

Description of Additional Supplementary Files

Supplementary Data 1. Differential expression analysis of ZFR knockdown in HEK-293TO cells
DESeq2 analysis of genes differentially expressed in RNAseq of HEK-293TO cells depleted of ZFR or treated with control siRNAs.

Supplementary Data 2. MISO analysis of alternative splicing induced by ZFR knockdown in HEK-293TO cells

Complete MISO output comparing splicing events in HEK-293TO cells depleted of ZFR or treated with control siRNAs.

Supplementary Data 3. MISO analysis of alternative splicing induced by ZFR knockdown in THP-1 cells

Complete MISO output comparing splicing events in monocytic (untreated) or macrophage-like (PMA-treated) THP-1 cells depleted of ZFR or treated with control shRNAs.

Supplementary Data 4. Differential expression analysis of ZFR knockdown in THP-1 cells
DESeq2 analysis of genes differentially expressed in RNAseq of monocytic (untreated) or macrophage-like (PMA-treated) THP-1 cells depleted of ZFR or treated with control shRNAs..

Supplementary Data 5. GSEA analysis of THP-1 ZFR knockdown RNAseq

Complete GSEA output from analysis of genes regulated by ZFR depletion from macrophage-like THP-1 cells.

Supplementary Data 6. Oligonucleotide sequences. Sequences of oligonucleotides used for cloning and gene expression analysis by RT-PCR.
